# Multilevel analysis of undernutrition and associated factors among adolescent girls and young women in Ethiopia

**DOI:** 10.1186/s40795-022-00603-x

**Published:** 2022-09-19

**Authors:** Wubshet Debebe Negash, Samrawit Mihret Fetene, Ever Siyoum Shewarega, Elsa Awoke Fentie, Desale Bihonegn Asmamaw, Rediet Eristu Teklu, Fantu Mamo Aragaw, Daniel Gashaneh Belay, Tewodros Getaneh Alemu, Habitu Birhan Eshetu

**Affiliations:** 1 Department of Health Systems and Policy, Institute of Public Health, College of Medicine and Health Sciences, University of Gondar, P.O. Box: 196, Gondar, Ethiopia; 2 Department of Reproductive Health, Institute of Public Health, College of Medicine and Health Sciences, University of Gondar, Gondar, Ethiopia; 3 Department of Epidemiology and Biostatistics, Institute of Public Health, College of Medicine and Health Sciences, University of Gondar, Gondar, Ethiopia; 4 Department of Human Anatomy, College of Medicine and Health Sciences, University of Gondar, Gondar, Ethiopia; 5 Department of Pediatrics and Child Health Nursing, School of Nursing, College of Medicine and Health Sciences, University of Gondar, Gondar, Ethiopia; 6 Department of Health Education and Behavioral Sciences, Institute of Public Health, College of Medicine and Health Sciences, University of Gondar, Gondar, Ethiopia

**Keywords:** Undernutrition, Adolescent girls and young women, Factors; Multilevel, Ethiopia

## Abstract

**Background:**

The consequences of undernutrition have serious implication for the health and future reproductive periods of adolescent girls and young women aged 15–24 years. Inspite of this, they are neglected age groups and there is limited information about the nutritional status of this age group in Ethiopia. Therefore, estimating the extent and associated factors of undernutrition among adolescent girls and young women in a national context using multilevel analysis is essential.

**Methods:**

Secondary data analysis was conducted from the Ethiopian Demographic and Health Survey 2016. A total sample weight of 5362 adolescent girls and young women was included in this study. A multilevel mixed-effect binary logistic regression model with cluster-level random effects was fitted to determine the associated factors of undernutrition among adolescent girls and young women in Ethiopia. Finally, the odds ratios along with the 95% confidence interval was generated to determine the individual and community level factors of undernutrition. A *p*-value less than 0.05 was declared as the level of statistical significance.

**Results:**

Overall, 25.6% (95%CI: 24.5–26**.**9) of adolescent girls and young women were undernourished. Statistically significant individual level factors includes adolescent girls and young women aged 15–19 years (AOR: 1.53, 95%CI: 1.32–1.77), individual media exposure (AOR: 0.82, 95%CI: 0.69–0.97), and unprotected drinking water source (AOR: 1.24, 95%CI: 1.04–1.48). Whereas, Southern Nations, Nationalities, and Peoples' Region (AOR: 0.33, 95%CI: 0.13–0.83) and rural residence (AOR: 1.69, 95%CI: 1.24–2.32), were community level factors for adolescent girls and young women undernutrition.

**Conclusion:**

One quarter of the Ethiopian adolescent girls and young women were undernourished. Therefore, the Ethiopian government should better engage this age group in different aspects of the food system. To improve nutritional status, public health interventions such as increased media exposure for rural residents and interventions that improve access to protected water sources will be critical.

## Introduction

The World Health Organization (WHO) defined adolescent girls and young women (AGYW) as those females under the age category of 15–24 years and makeup about 20% of the world’s population and are characterized by significant physiological, psychological, and social changes that place their lives at high risk [1, 2]. Nutritional requirements like protein, carbohydrates, and others are required in increased amounts during this age [3].

Worldwide, nutritional deficiencies, suboptimal linear growth, and undernutrition are major public health problems [3]. Globally, the prevalence of stunting, wasting and underweight are 29.1%, 6.3%, and 13.7%, respectively [4]. In particular, the sub-Saharan Africa region is more affected by undernutrition among AGYW, in which 13.7% and 12% are stunted and wasted in SSA as compared to 5.2% and 10.4% in Europe and central Asia are stunted and wasted, respectively [5].

Undernutrition had multiple consequences like anemia, delayed growth, retarded intellectual development, increase infection, inadequate bone mineralization, and long-term effects like stillbirths, complicated delivery, and maternal death in the future lives of individuals [3, 6–8].

Studies in Ethiopia showed that the magnitude of undernutrition is different among pregnant mothers and adolescent girls. For example among pregnant mothers rates, ranged from 14.4% in Gondar [9] to 19.5% in Dessie [10]. In addition, 29.2% of adolescent girls were stunted, and 30.4% were wasted [11, 12]. Another systematic review in Ethiopia also revealed that the pooled prevalence of stunting and underweight among adolescent girls were 20.7% and 27.5%, respectively [7].

Large family size, rural residence, unprotected source of drinking water, lack of latrine, low dietary diversity score, mother illiteracy, and food insecure households were identified as contributing factors to adolescent undernutrition in the literature [7, 13].

To overcome the aforementioned problems, multiple nutritional strategies and political support for nutritional programs are essential methods for improving the nutritional status of all reproductive aged women [14, 15].

There has been significant reduction in undernutrition in Ethiopia over the past two decades (from 30 in 2000 to 22 in 2016 for women) and has been implementing a nutrition focused agenda to catalyze improvements in nutrition corresponding with the UN Decades of Action on Nutrition (2025) and the Sustainable Development Goals (2030) [16]. Moreover, the country recently approved a new food and nutrition policy such as increasing agricultural production and productive safety net programs in an effort to reduce undernutrition [17, 18].

Even though nutrition interventions focused on children and pregnant women, the conception of protein-rich foods and micronutrient supplementations, and the prioritization of AGYW aged 15–24 years was relatively neglected in research priority. Many studies have tried to address undernutrition among under-five and adolescent girls [1, 19–23]. Therefore, this study tried to assess the prevalence of undernutrition and associated factors among AGYW in Ethiopia by considering both the individual and community level factors. Once the nutritional status of AGYW is known, interventions aimed at the nutritional finding have shown improvements in birth weight, preterm delivery, and negative long-term outcomes [24, 25]. Thus, identifying the prevalence and associated factors of undernutrition among AGYW is very crucial, and the evidence found from the study will help policymakers, governments, and stakeholders to have an insight to design context-specific interventions.

## Methods and materials

### Study setting

Ethiopia is found in the horn of Africa, and is administratively divided into nine ethnically, and politically autonomous regional states (Tigray, Afar, Amhara, Oromia, Benishangul Gumuz, Gambela, Southern Nations Nationalities and People Region, Harari, and Somali) and two administrative cities (Addis Ababa and Dire-Dawa). The regions are administratively divided into zones, and zones into districts (the third administrative division). With an estimated population of 118 million, Ethiopia is the 14^th^ most populous country in the world and the second most populous on the African continent [26].

### Study design and period

A community-based cross-sectional survey was conducted in 2016 among reproductive age women in Ethiopia. The 2016 EDHS is the fourth survey conducted from January 18 to June 27/2016 in Ethiopia. The Ethiopian Demographic, and Health Survey (EDHS) is a national-level study conducted every five years as part of the worldwide Demographic Health Survey (DHS) [23, 27].

### Population and eligibility criteria

The AGYW during the survey in Ethiopia were the source population, while the study population were all the AGYW who were in the selected enumeration areas included in the analysis. Whereas pregnant, postpartum, AGYW who gave birth in the two months preceding the date of the interview were excluded from the analysis because it affects the estimate of BMI as weight changes during pregnancy and postpartum periods.

### Data source and sampling procedure

The 2007 Ethiopian population and housing census, which was conducted by the central statistical agency (CSA) of Ethiopia was used as a sampling frame for the 2016 EDHS. A total of 645 enumeration areas (EAs) (202 urban and 443 rural) were used for the census. The EDHS employed two stage stratified-cluster sampling technique. Proportional allocation was achieved within each sampling stratum before sample selection at different levels. In the first stage, 645 EAs were selected with a probability proportional to EAs size, and each sampling stratum was selected from the given samples. Household listing operations were implemented to determine the number of residential units in each EA. Then, the resulting lists of households were used as the sampling frame for selecting households. In the second stage, 28 households from each cluster (645) were selected with an equal probability. Interviews were conducted only with households that had been preselected. More detailed information about the methodology or sampling design is available in EDHS 2016 report [28]. All adolescent girls and young women aged 15–24 years who are the usual members of selected households and visitors who slept in the household the night before the survey were eligible for the survey [23].

### Study variables

The dependent variable of the study was undernutrition among AGYW, which was measured by weight (Kg) and height (m^2^) reports of Body Mass Index (BMI). Therefore, in this study undernutrition was defined as less than 18.5 kg/m^2^ (which includes either stunting or underweight) [29, 30]. The effect of factors on the outcome variable (undernutrition) for the i^th^ adolescent girls and young women in the j^th^ cluster (Yij) was dichotomized as follows: Yij = 1 if BMI < 18.5 kg/m^2^ as undernutrition, and 0 if BMI ≥ 18.5 kg/m as not undernutrition.

Individual level factors (level-one) included AGYW age, educational status, religion, number of family members, individual level media exposure**,** and source of drinking water, while community level factors (level-two) included region, residence, community level media exposure, community level poverty, and community level literacy. The aggregate community level independent variables (community level poverty, community level media exposure, and community level literacy) were constructed by aggregating individual-level characteristics at the community (cluster) level. They were categorized as high or low based on the distribution of the summary of the proportion values calculated after checking the distribution using the histogram for each variables. In the aggregate variable, there was no normal distribution, so the median value was used for categorization. And finally, model three (level three) examined both individual and community-level variables simultaneously.

### Data management and analysis

The data extraction, cleaning, recoding, and labeling for further analysis were done using STATA version 14 statistical software and Microsoft Excel.

Age was grouped as 15–19 and 20–24. Occupation was coded as employed, sales/merchant, agriculture, and others. No formal education, and formal education were the categories for educational level of the women. Wealth index was recoded as poor, middle and rich. Media exposure was coded as ‘yes’ for those AGYW who had read newspapers/magazines or listening radio and/or watching television less than once a week/at least once a week and otherwise ‘no’. < 5 and ≥ 5 were codes for family size. Source of drinking water categorized as protected and unprotected. Community level variables (community level media exposure, community level poverty and community level literacy) were generated by aggregating the individual level factors at cluster level and categorized them as high if the proportion is ≥ 50% and low if the proportion is < 50% based on the national median value since these were not normally distributed [31].

Before analysis sampling weight of each variable was done to restore the unequal probability of selection between strata to get reliable estimates. Out of 15,683 total eligible households, 6,401 were AGYW aged 15–24 years. Of this, 418 and 505 AGYW who were currently pregnant and gave birth in the two months preceding the date of the interview were excluded, respectively. Lastly, 5,478 AGYW were included in the analysis. Overall, a total weighted sample of 5,362 AGYW were included in this study.

The second step was a bivariable analysis that calculated the proportion of undernutrition across the independent variables with their *p*-values. All the variables having a *p*-value less than 0.2 in bivariable analysis were used for multivariable analysis. For the multivariable analysis, adjusted odds ratios with 95% confidence intervals and a *p*-value of less than 0.05 were used to identify associated factors of undernutrition. In the final step of the analysis, a multilevel logistic regression analysis comprising fixed effects and random effects was done.

Data eligibility for multilevel analysis was checked before analysis (Intra-class Correlation Coefficient (ICC) greater than 10% (ICC = 12.2%)). As a result of the hierarchical nature of EDHS data, adolescent girls and young women are nested within communities (clusters). In this case, the assumptions of independence and equal variance in a logistic regression model may not be met. Therefore, a multilevel binary logistic regression model was used to estimate the effect of individual and community-level variables on undernutrition [32]. Four models were fitted for multilevel analysis; null model (model 0) which shows the variations in undernutrition in the absence of any independent variables. Model I an adjusted for the individual-level variables, Model II adjusted for the community level variables, and model III adjusted for both individual and community level variables. Simultaneously, model fitness was done using the deviance (-2 log likelihood). The results of the fixed effects of the model were presented as adjusted odds ratio (AOR) while the random effects were assessed with intra-class correlation coefficient (ICC). Variance inflation factor (VIF) was used to check for multicollinearity among independent variables and it was found no multicollinearity (mean value for the final model = 1.87) (Table 4).

## Results

### Socio-demographic characteristics of study participants

A total of 5362 AGYW were included in the final analysis. The median age of the study participants was 19 (IQR: 17–24) years. More than half (57.6%) of the study participants were under the age group of 15–19 years. For 55.8 percent of the population, the family size was less than five people (Table 1).

**Table 1:** Sociodemographic characteristics of study participants in Ethiopia, 2016 (*n* = 5362**)**

Variables	Categories	Frequency(n)	Percentage (%)
Age of respondents	15–19	3088	57.6
	20–24	2274	42.4
Religion	Orthodox	2396	44.7
	Muslim	1550	29.0
	Protestant	1310	24.4
	Catholic	49	0.9
	Traditional	38	0.7
	Others^a^	19	0.3
Household wealth index	Poor	1672	31.2
	Middle	992	18.5
	Rich	2698	50.3
Educational status of the respondent	No education	991	18.5
	Primary	2948	55
	Secondary	1063	19.8
	Higher	360	6.7
Occupation	Employed	2971	55.4
	Sales/merchant	155	2.9
	Agricultural	690	12.9
	Skilled/ manual	1127	21
	Others ^a^	419	7.8
Family size	< 5	2993	55.8
	≥ 5	2369	44.2
Individual level media exposure	Yes	2594	48.4
	No	2768	51.6

^a^no work, clerical, unskilled manual

### Community-level variables

The majority (75.5%) were rural dwellers. More than half (51.6%) had community-level media exposure (Table 2).

**Table 2:** Community-related characteristics of participants in Ethiopia, 2016 (*n* = 5362)

Variables	Categories	Frequency(n)	Percentage (%)
Residence	Urban	1315	24.5
	Rural	4047	75.5
Region	Tigray	437	8.1
	Afar	47	0.9
	Amhara	1275	24
	Oromia	1893	35.3
	Somali	140	2.6
	Benshangul Gumuz	55	1.0
	SNNPR^a^	1082	20.2
	Gambela	16	0.3
	Harari	13	0.2
	Addis Ababa	373	6.9
	Dire Dawa	31	0.5
Community level media exposure	Low	3177	59.3
	High	2185	40.7
Community level literacy	Low	2979	55.6
	High	2383	44.4
Community level poverty	Low	2519	47.0
	High	2843	53.0

^a^Southern Nations, Nationalities, and Peoples' Region

### Undernutrition status

The prevalence of undernutrition among AGYW in Ethiopia was 25.6% (95% CI: 24.5–26.9).

### Individual and community level factors of undernutrition (fixed-effects)

In the final model (Model III) after adjusting for individual and community level factors, Individual level variables such as age of AGYW, individual media exposure, source of drinking water, educational level of AGYW, family size, individual level wealth index and from the community level variables, region, residence, community level poverty, community level media exposure and community literacy were candidate variables for multivariable logistic regression analysis. From the aforementioned variables, age of AGYW, individual level media exposure, source of drinking water, region, and residence were significantly associated variables with undernutrition among AGYW.

Correspondingly, the odds of undernutrition among AGYW aged 15–19 years were 1.53 times higher than those AGYW aged 20–24 years (AOR: 1.53, 95%CI: 1.32–1.77). AGYW having individual media exposure had 18% less odds to develop undernutrition than those who had no media exposure (AOR: 0.82, 95%CI: 0.69–0.97). The odds of undernutrition among AGYW who live in the Southern Nations, Nationalities, and Peoples' Region were 67% less likely than AGYW who live in Dire Dawa (AOR: 0.33, 95%CI: 0.13–0.83). The odds of undernutrition among rural resident AGYW were 69% times higher than those AGYW who reside in urban (AOR: 1.69, 95%CI: 1.24–2.32). Those AGYW who had used unprotected drinking water sources were 1.24 times more likely to develop undernutrition than those AGYW who had protected drinking water source (AOR: 1.24, 95%CI: 1.04–1.48) (Table 3).

**Table 3:** Multi-level mixed-effect logistic regression analysis of factors associated with undernutrition among AGYW, EDHS 2016 (*n* = 5362)

**Variables**	**Categories**	**Undernutrition**		Model 1 AOR (95% CI)	Model 2 AOR (95%CI)	Model 3 AOR (95%CI)
		Yes n (%)	No n (%)			
Age in years	15–19	897(29)	2191(71)	1.6(1.4–1.8)		1.53(1.32–1.77)*
	20–24	481(21.15)	1793(78.85)	1		1
Media exposure	Yes	646(23.32)	2122(76.68)	0.8(0.68–0.94)		0.82(0.69–0.97)*
	No	732(28)	1862(72)	1		1
Mothers education	Formal education	1101(25)	3270(75)	0.98(0.81–1.19)		1.03(0.84–1.25)
	No formal Education	277(27)	714(72)	1		1
Family size	< 5	730(24.4)	2263(75.6)	1		1
	> = 5	647(27.32)	1722(72.68)	1.08(0.94–1.25)		1.07(0.93–1.23)
Source of drinking water	Protected	268(21.96)	950(78.04)	1		1
	Unprotected	1110(26.8)	3034(73.2)	1.26(1.05–1.51)		1.24(1.04–1.48)*
Wealth index	Poor	484(28)	1188(71)	1		1
	Middle	301(30.4)	690(69.6)	1.08(0.89–1.32)		1.14(0.93–1.39)
	Rich	592(22)	2106(78)	0.78(0.65–0.94)		0.85(0.69–1.05)
Region	Dire Dawa	8(26)	23(74)		1	1
	Tigray	174(39.8)	263(60.2)		1.46(0.60–3.51)	1.69(0.67–4.27)
	Afar	21(44.7)	26(55.3)		1.95(0.68–5.61)	1.86(0.64 s-5.47)
	Amhara	363(28.5)	911(71.5)		0.78 (0.33–1.88)	0.86(0.35–2.34)
	Oromia	509(27)	1384(73)		0.70(0.29–1.68)	0.75(0.31–1.87)
	Somali	59(11.7)	82(88.3)		1.54(0.60–3.94)	1.41(0.54–3.65)
	Benshangul Gumuz	11(20)	44(80)		0.50(0.17–1.50)	0.54(0.18–1.68)
	SNNPR	148(13.7)	934(86.3)		0.29(0.12–0.72)	0.33(0.13–0.83)*
	Gambella	6(33.9)	11(66.1)		1.28(0.33–5.04)	1.51(0.38–6.0)
	Harari	3(27.7)	9(72.3)		1.03(0.22–4.80)	1.09(0.23–5.17)
	Addis Ababa	76(20.4)	297(79.6)		0.84(0.34–2.06)	0.97(0.39–2.44)
Community level poverty	Low	600(23.8)	1919(76.2)		1	1
	High	778(27.4)	2065(72.6)		0.91(0.72–1.14)	0.79(0.62–1.02)
Community level media exposure	Low	875(27.5)	2302(72.5)		1	1
	High	503(23.0)	1682(77.0)		0.78(0.61–1.00)	0.86(0.66–1.11)
Community literacy	Low	745(25)	2234(75)		1	1
	High	632(26.5)	1751(73.5)		1.24(1.00–1.54)	1.18(0.62–1.48)
Residence	Urban	255(19.4)	1060(80.6)		1	1
	Rural	1123(27.7)	2924(72.3)		1.89(1.40–2.56)	1.69(1.24–2.32)*

*AOR* Adjusted Odds Ratio, *COR* Crude Odds Ratio, *Null model* adjusted for individual-level characteristics, *Model2* adjusted for community-level characteristics, *Model 3* adjusted for both individual and community-level characteristics

*Statistically significant at *p*-value < 0.05

### Random effects (measures of variation)

There was a significant variation in the prevalence of undernutrition among AGYW across the clusters. The intra-cluster correlation coefficient (ICC) for the null model was 12.2%. This means that 12.2% of the variation in undernutrition among AGYW is due to differences in regions/clusters (between cluster variations). Model comparison was employed using deviance. Correspondingly, the model with the lowest deviance was selected. To identify associated factors of undernutrition among AGYW, an adjusted odds ratio with a 95% confidence interval was calculated. In the multivariable analysis, a *p*-value of 0.05 was used to declare statistical significance of the association. The median odds ratio (MOR) showed undernutrition was heterogeneous among clusters. In the first model, the value of MOR was 1.9. Which implied that AGYW within the cluster of higher undernutrition had a 1.9 times higher chance of undernourishment than AGYW within a cluster of lower undernutrition if AGYW were randomly selected from different clusters (EAs). Concerning PCV, 50% of the undernutrition variability was explained by the final model (Table 4).

**Table 4:** A measure of variation for undernutrition among AGYW at cluster level by multilevel binary regression analysis, EDHS 2016 (*n* = 5362)

Measures of variation	Null model	Model 1	Model 2	Model3
Variance	0.46	0.38	0.25	0.23
MOR	1.9(1.73–2.12)	1.8	1.61	1.6
PCV (%)	Reference	17.4	45	50
ICC (%)	12.2	10.6	7.1	6.8
Model fitness				
Deviance(-2×Likelihood)	5957.4	5815.4	5814.8	5736.6
**Mean VIF**	…	1.22	2.04	1.87

*ICC* Intra-class correlation coefficient, *MOR* Median odds ratio, *PCV* Proportional Change in Variance, *Null model* without independent variables, *Model 1* only individual-level variables, *Model 2* only community-level variables, *Model 3* both individual and community-level variables, *VIF* Variance Inflation Factor

## Discussion

Globally, one third of the population suffers from malnutrition [33]. Especially in low and middle income countries, women are highly vulnerable to all forms of malnutrition [34, 35]. There is no doubt that AGYW nutrition plays a crucial role in the development of maternal, newborn, and child health [36]. It is imperative that preventable determinants of undernutrition be identified and reduced in order to improve the world’s commitment to end malnutrition by 2030 [34].

This study carried out to assess the prevalence and associated factors of undernutrition among AGYW in Ethiopia. Based on this, one-fourth, 25.6% (95% CI: 24.5–26.9), of AGYW in Ethiopia had undernourished. This is in line with the research report 26.4% conducted in southern Ethiopia [37]. This finding is higher than studies conducted in Gondar, Ethiopia in which 14.4% were undernourished [9]. Similarly, the finding is higher than studies conducted among adolescent girls, which is 20.7% [7, 38] and the 19.06% among pregnant women reported in southern Ethiopia [39]. The possible justification for this difference might be the difference in the study participants. In this study the study participants were AGYW while the other studies were among adolescent girls and pregnant mothers. Furthermore, variations in the magnitude of undernutrition may be explained by differences in data source and sample size.

The magnitude of undernutrition is lower than a study conducted in Ethiopia, where 29.2% and 30.4% adolescent girls were wasted and stunted, respectively [11, 12]. Similarly this study is lower than the 43.8% study report among rural pregnant women in eastern Ethiopia [38]. This is most likely due to a difference in the source population, as well as differences in the study period, sampling technique, and size. The above studies were conducted as primary research, whereas in our study we used secondary data sources. The lower prevalence might also be accounted for by the governments ongoing improvement to reduce undernutrition [17, 18].

The outcome was attributed to both individual and community level factors. Youth age, individual media exposure, unprotected drinking water source, occupational status, region, and residence were significant associated factors of undernutrition among AGYW after adjusting for individual and community level variables.

Accordingly, the current study identified that AGYW, aged 15–19 years were 1.53 times more likely to develop under nutrition than those aged 20–24 years. This is supported by studies in Ethiopia where early adolescent were two times more likely to develop undernutrition than late adolescents [40]. The same holds true in India [41] and Nigeria [42]. This might be due to the reason that the effect of growth velocity synergistic, meaning that there is a synergistic effect of growth velocity during puberty when peak height velocity occurs [43]. In addition, as age increases linear growth is also marked by the lengthening of long bones at the growth plate followed by epiphyseal closure when growth is completed [44]. Moreover, lower-aged girls probably often have little power in decision-making about food in the household. This indicates that nutritional strategies and political support for nutritional programs are essential methods for improving the nutritional status, focusing on the lower age group.

Those who had media exposure had an 18% lower odds of being undernourished than those who had no media exposure. This is in line with another study in Ethiopia [44]. This could probably be those who had access to the media (radio, newspapers, and television) could get information about a balanced diet, the importance of a variety of foods and health programs. So, increasing media exposure of health programs has a role in overcoming undernutrition among AGYW.

The odds of undernutrition, among AGYW, in the region of SNNPR were 67% lower compared to Dire Dawa city. This is in agreement with another study in Ethiopia [45]. This might be differences in socio-demographic and economic status, and the availability of divergent foods in the SNNPR.

This study revealed that the odds of undernutrition among AGYW from rural residents was nearly 70% higher compared with urban residents. This is in line with another study in Ethiopia, Ghana, and Tanzania [7, 46–48]. The possible reason might be their educational status as most rural residents are illiterate, which is associated with the inaccessibility of information about medical issues for rural residents. Food security in rural areas depends on natural and human resources that are vulnerable to change including rain or weather patterns, agricultural knowledge and human capital [49]. So, nutritional education like a balanced diet and food security are needed for rural residents like AGYW.

The current study identified that AGYW who had used an unprotected drinking water source were 1.24 times more likely to become undernourished compared with those who had used a protected water source. The finding is in line with other studies conducted in Ethiopia [7, 11]. This could be due to the fact that an unprotected source of drinking water leads to communicable diseases, bacteria, and intestinal parasites resulting in micronutrient depletion and finally leadings to undernutrition [50, 51].

### Strengths and limitations of the study

For this study the following strengths and limitations are forwarded; its large sample size, nationally representative data. This study also employed a multilevel-modeling technique to identify a more valid result that takes the survey data’s hierarchical nature into account. Furthermore, the DHS methodology allows for comparison with other settings. However, because the data is cross-sectional, the temporal relationship of causations cannot be established.

## Conclusions

One quarter of the Ethiopian AGYW were undernourished. Age group 15–19 years, individual media exposure, region, rural residents, and unprotected drinking water sources were significant associated factors for AGYW undernutrition. Therefore, considering the intergenerational effect of undernutrition, the Federal Ministry of Health (FMOH) should increase media exposure, particularly for rural residents. And the Ethiopian government needs to better engage this age group in different aspects of food systems. Moreover, improve access to protected water sources for enhancing the safety of drinking water is an important intervention.

## Data Availability

The data used in this study were publicly available. The data set can be found in the following website: https://dhsprogram.com/data/dataset/Ethiopia_Standard-DHS_2016.cfm.

## Abbreviations

AGYW: Adolescent girls and Young Women
AOR: Adjusted Odds Ratio
BMI: Body Mass Index
DHS: Demographic Health Survey
EAs: Enumeration Areas
EDHS: Ethiopian Demographic and Health Survey
ICC: Intra-class Correlation Coefficient
MOR: Median Odds Ratio
PCV: Proportional Change in Variance
SD: Standard Deviation
SNNPR: Nations, Nationalities, and Peoples' Region
WHO: World Health Organization
